# Unity Is Strength: The Mutual Alliance between CFTR and SLC26A6 as Therapeutic Opportunity in Cystic Fibrosis

**DOI:** 10.3390/ph17030367

**Published:** 2024-03-12

**Authors:** Marilena Pariano, Cinzia Antognelli, Luigina Romani, Claudio Costantini

**Affiliations:** Department of Medicine and Surgery, University of Perugia, 06132 Perugia, Italy; marilena.pariano@gmail.com (M.P.); cinzia.antognelli@unipg.it (C.A.); luigina.romani@unipg.it (L.R.)

**Keywords:** cystic fibrosis, cystic fibrosis transmembrane conductance regulator, SLC transporters, protein–protein interactions

## Abstract

Patients with cystic fibrosis (PwCF) have recently experienced an unprecedented breakthrough with the adoption of modulator therapy in clinical practice. This remarkable achievement has led to the reconsideration of disease management as the increased life expectancy has gradually shifted the attention over a spectrum of extra-pulmonary manifestations that become prevalent in the aging population. It comes to be that complementary approaches that target patient co-morbidities are needed for the optimal clinical management of PwCF. A strategy would be to adjuvate the cystic fibrosis transmembrane conductance regulator (CFTR) in performing its functions in the different organs in which it is expressed. Solute carrier family 26 (SLC26) members appear ideal in this context. Indeed, they not only cooperate with CFTR in the organ-dependent regulation of ion fluxes but physically interact with it to reciprocally modulate their function. In this opinion, we summarize available evidence pointing to a physical and functional interaction between CFTR and SLC26 members, with a particular focus on SLC26A6 for its wider expression and broader anion selectivity, and then discuss how restoring the physical interaction between CFTR and SLC26A6 might be beneficial in the treatment of PwCF in the era of modulator therapy.

## 1. Introduction

Cystic fibrosis (CF) is an autosomal recessive disorder affecting at least 100,000 people worldwide [1]. It is caused by mutations in the gene encoding for the cystic fibrosis transmembrane conductance regulator (CFTR), an ion channel that regulates the ionic content of luminal fluid [2]. The unrelenting decline in respiratory function has typically represented the major cause of morbidity and mortality in patients with CF (PwCF). The efforts to delay or prevent the progressive course of the disease have been recently rewarded by the approval of Trikafta, a highly effective modulator therapy (HEMT), which targets the most common F508del mutation with profound effects on prognosis and quality of life [3]. The increased life expectancy of PwCF has opened up a novel perspective on disease management as patients transition from childhood into adulthood [4]. Indeed, although traditionally regarded as a chronic respiratory disease, CF affects multiple organs, such as the gastrointestinal and the reproductive tracts, and exposes the patients to a plethora of metabolic complications, including, among others, CF-related diabetes and CF-related bone disease or osteoporosis [2], that may become predominant in the aging population. Even more so, the effects of Trikfta against extra-pulmonary manifestations of the disease, such as the gastrointestinal (GI) symptoms [5], are still debated.

The complex clinical presentation of PwCF reflects the tissue-specific expression pattern [6] and regulation [7] of CFTR in the human body and its functional role in regulating liquid homeostasis in the different compartments. This activity is performed in association with other ion channels, with tissue-specific or ubiquitous expression, and is finely tuned to meet the physiological requirements of each compartment in a context-dependent manner. This is important not only in an etiopathological perspective but also in terms of the variable individual response to corrector therapy, upon which restoring CFTR expression and function should occur in harmony with co-expressed ion channels to preserve tissue physiology. In this regard, recent interactome studies aimed at identifying CFTR protein partners have revealed a multiplicity of proteins [8], including multiple solute carrier (SLC) transporters [9], suggesting that ion channels are not only reciprocally influenced by ions movement but also by direct, physical interactions, the quantitative characterization of which [10] may inform on the extent of regulation of CFTR function by single protein–protein interactions [11].

SLC transporters represent the largest membrane transport group in humans, with 455 members classified into 66 families, and mediate the transport of a broad range of solutes, including nutrients, neurotransmitters, ions, and drugs, across biological membranes, participating in numerous biological processes [12]. Among the various families, the SLC26 gene family encodes anion exchangers and anion channels able to transport a variety of substrates, including Cl^−^, HCO_3_^−^, sulfate, oxalate, I^−^, and formate [13]. SLC26 and the related SulP (sulfate permease) genes have been described in nearly all organisms studied from archaea, bacteria, fungi, plants, and animals. The mammalian SLC26 gene family comprises 11 genes, i.e., SLC26A1–A11 [13]. Structurally, SLC26 proteins show a short cytoplasmic N-terminal region followed by a transmembrane domain with 10–14 times putative membrane-spanning α-helices, and the C-terminal cytoplasmic region, that includes the sulfate transporter and anti-sigma factor antagonist (STAS) domain [13]. Intriguingly, the STAS domain of SLC26 proteins binds to the regulatory (R) domain of CFTR, a process facilitated by their PDZ domains, in turn promoting the assembly of a multi-component complex [14]. Of interest, the binding of STAS and R domains are enhanced by phosphorylation of the R domain by protein kinase A (PKA) [14], such that the interaction of CFTR with SLC26 members is linked to its functional activation. This finding has allowed us to appreciate a deeper connection between CFTR and SLC26 protein members, with implications for CF etiopathogenesis as well as the identification of novel therapeutic approaches.

In this opinion, we will focus on one of the SLC26 members, i.e., SLC26A6, characterized by wide tissue expression, including the pancreas, the intestine, and the kidney, and broad anion selectivity, ranging from halides to monovalent and divalent oxyanions, thus being potentially involved in multiple manifestations of the disease. In parallel, we will present available evidence on the tissue-specific interactions between SLC26A6 and CFTR and on how this cross-talk reverberates on their respective functions. Finally, we will translate this paradigm in the current era of CFTR correctors aimed at restoring CFTR expression and function by highlighting the need for maintained interactions with SLC26 family members to preserve ionic regulation of luminal fluids in the distinct compartments impacted by the disease.

## 2. SLC26A6 and CFTR: A Mutual Alliance

SLC26A6 was identified in the early 2000s and first characterized for its abundant expression in the kidneys and pancreas [15,16]. The mouse orthologue was identified shortly after, shown to be expressed on the brush border membrane of renal proximal tubule cells and characterized as a chloride-formate exchanger [17]. It was then shown that rodent SLC26A6 may also work as chloride-bicarbonate exchanger in the villus cells of the duodenum [18], gastric parietal cells [19] as well as rat kidney proximal tubules [20]. Subsequent studies revealed that SLC26A6 has also affinity for oxalate and sulfate and can work in multiple exchange modes [21,22]. In this regard, it should be noted that mouse and human SLC26A6 present differences in transport activity and regulation but can both mediate chloride-bicarbonate exchange [23,24]. Functional consequences of SLC26A6 deficiency were assessed upon generation and characterization of Slc26a6-null mice, revealing a major contribution in the kidney proximal tubule (chloride/base exchange and oxalate-stimulated NaCl absorption) [25], in the duodenum (bicarbonate secretion) [25,26], in the ileum (mucosal-to-serosal oxalate flux) [27,28], and in pancreatic ducts (bicarbonate secretion) [29].

In an attempt to identify the mechanisms responsible for the defect in HCO_3_^−^ transport in CF, it was shown that CFTR could upregulate SLC26A3 and SLC26A6 expression in cultured pancreatic duct cells [30] as well as stimulate their activity [31], thus providing a first indication of the potential cross-talk between CFTR and SLC26 family members. Structurally, SLC26A6 contains an even number of transmembrane domains such that the N- and C-termini are located intracellularly [32]. Notably, the C-terminus contains a PDZ interaction motif identical to CFTR and, similar to the latter, able to bind to the Na^+^/H^+^ exchanger (NHE)3 kinase A regulatory protein (E3KARP) and NHE3 regulatory factor (NHERF) proteins, raising the interesting hypothesis that a multiprotein complex made up of NHERF, E3KARP, SLC26A6, and CFTR might form within the cell [32]. This proved to be true, as the PDZ ligands facilitated the direct interaction between SLC26A6 (and SLC26A3) and CFTR mediated by their STAS and R domains, respectively, leading to activation of both transporters [14,33].

**Figure 1:**
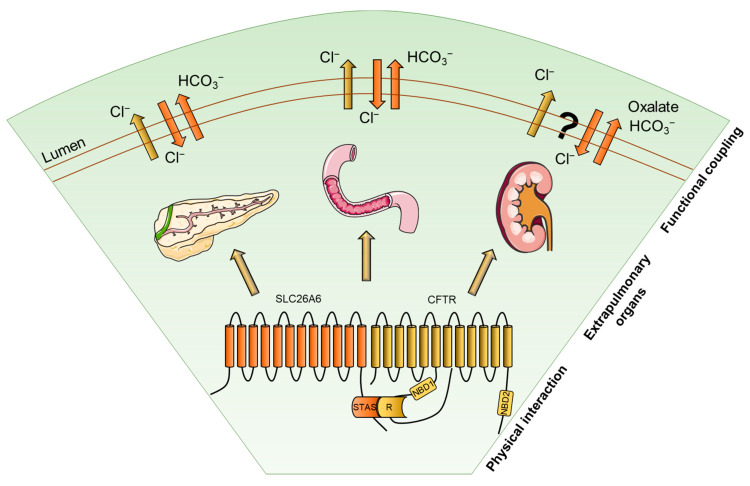
Physical interaction and functional coupling of CFTR and SLC26A6 in extrapulmonary organs. The picture depicts CFTR and SLC26A6 interacting via the R and STAS domains, respectively, and their functional coupling in pancreas, duodenum, and kidney. Details are described in the text.

Models of CFTR–SLC26A6 interaction have been investigated in several tissues, such as the pancreas and the intestine, each characterized by specific chloride-bicarbonate physiological requirements (Figure 1).

In the pancreas, the duct secretes bicarbonate at a high concentration (up to 140 mM) while absorbing most of the chloride [34]. It has been hypothesized that the SLC26 members SLC26A6 and SLC26A3, mainly distributed in the proximal and distal ducts, respectively, and CFTR concur to the formation of the pancreatic juice. Specifically, in the proximal tube, CFTR secretes chloride while SLC26A6 contributes to bicarbonate secretion and chloride absorption [35]. Indeed, activation of CFTR by forskolin stimulates bicarbonate secretion, but only in the presence of Slc26a6 [36]. As a result of SLC26A6 and CFTR activity, the concentration of chloride decreases while bicarbonate levels increase as the fluid moves along the duct. Finally, in the distal duct, bicarbonate secretion is mediated by SLC26A3 and CFTR [35,37].

In the intestine, SLC26A6 is predominantly located in the duodenum, in which bicarbonate secretion is crucial to protect against the acidic chyme coming from the stomach [18]. Under basal conditions, the duodenal chloride-bicarbonate exchange is mediated by Slc26a6 and requires the presence of CFTR to provide a chloride leak [38,39]. Therefore, also in the intestine, a coupled activity of Slc26a6 and CFTR is involved in bicarbonate secretion, at least under basal conditions [40].

As previously mentioned, SLC26A6 is also involved in the transport of oxalate, an end product of metabolism whose accumulation in the body is the cause of hyperoxaluria, ultimately resulting in the formation of oxalate stones in the kidney and, in the most severe forms, systemically, with potential fatal outcomes [41]. SLC26A6 appears to be involved in secreting oxalate in the intestinal lumen. Indeed, slc26a6-knockout mice had reduced oxalate secretion in the duodenum and ileum and presented with hyperoxalemia [41]. Of note, cftr-/- mice showed reduced expression of SLC26A6 in duodenum and presented with oxalate levels in the serum and urine 2.5-fold higher than wild-type mice [42]. Thus, although not all studies are concordant [43,44], CF patients may be at higher risk for developing kidney stones, and this may become important in the aging population. Indeed, CF-related kidney disease is emerging as a critical aspect in aging PwCF [45]. While both SLC26A6 and CFTR are expressed in the kidney, no functional coupling for bicarbonate and/or oxalate transport has been reported.

## 3. Restoring SLC26A6 and CFTR Interaction in CF

As detailed above, the homeostatic regulation of the ionic content at different sites requires the coordinated activity of CFTR and SLC26A6, which, in turn, may be secondary to their physical interaction within a multiprotein complex. It is likely that the interaction between CFTR and SLC26 family members occurs within the endoplasmic reticulum (ER) along the secretory pathway, as recently shown for the lung-expressed SLC26A9 [46]. Most importantly, apical expression of SLC26A9 was reduced in cells expressing F508del–CFTR as a consequence of retention in the ER and enhanced proteasomal degradation and could be partially corrected by low temperature, administration of VX-809, or transfection with wild-type CFTR [46]. These results would indicate that restoring the positive interaction between CFTR and SLC26A9 may protect the latter from degradation and promote its apical expression. It is also worth noting that the degradation of SLC26A6 occurs via endoplasmic reticulum-associated degradation (ERAD) and is mediated by Hsp70-dependent targeting of the STAS domain [47], which is also implicated in the binding of the R domain of CFTR [14,33]. These findings raise the interesting hypothesis that promoting the interaction between SLC26A6 and CFTR via their STAS and R domains, respectively, might serve a dual role: first, it may prevent the ERAD-mediated degradation of SLC26A6, thus promoting its apical expression; second, it may facilitate the co-translocation of SLC26A6 and CFTR to the membrane and the restoration of their coupled activity. This strategy requires that F508del–CFTR be partially corrected by the use of modulators, thus favoring the positive interaction between CFTR and SLC26A6 over the mechanism driving the proteasomal degradation. An alternative or complementary approach might be represented by the use of protein–protein interaction (PPI) stabilizers designed to work at the interface of the STAS and R domains of SLC26A6 and CFTR, respectively. It is expected that forcing the interaction between the two molecules might work to overcome the ERAD-mediated degradation and force the exit of CFTR and SLC26A6 from the ER along the secretory pathway, irrespective of the use of modulators. The use of such stabilizers would therefore prevent CFTR and SLC26A6 retention and degradation in the ER, both effects concurring to their co-translocation to the membrane for ionic regulation.

**Figure 2:**
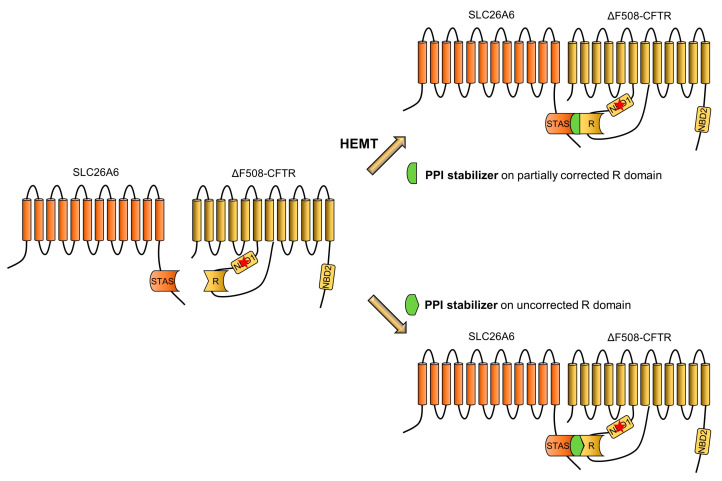
Restoration of physical interaction between CFTR and SLC26A6 upon administration of PPI stabilizers. The panel shows that the disrupted interaction between STAS and R domains in the F508-del mutation (indicated by a red star) (left side) can be restored by the use of PPI stabilizers in the presence (right side, upper panel) or absence (right side, lower panel) of HEMT. Details are described in the text. PPI: protein–protein interaction; HEMT: highly effective modulator therapy.

PPI stabilizers are increasingly being recognized as potential candidates in the development of novel drugs (see Box 1), and they work by means of two different mechanisms [48]. In the first mechanism, a compound works allosterically to change the conformation of one partner and increase its affinity for the other partner, while, in the second one, the compound binds directly at the interface between the two partners to stabilize their interaction [48]. In order to develop PPI stabilizers of the R and STAS domains of CFTR and SLC26A6, it is crucial to determine the extent to which the folding of the R domain is affected by the F508-del mutation [49]. Indeed, “in contrast to wild-type CFTR, in which the structured R domain inserts into a cytosolic cleft, little density corresponding to the R domain was visible in F508-del CFTR. As the R domain packs mainly along the surface of NBD1, it is possible that defects in NBD1 assembly also disrupt the correct positioning of the R domain” [50]. In particular, it is crucial to determine whether an interaction still occurs between the R and STAS domains or whether the misfolding completely prevents the interaction. In the latter case, it would be necessary to look for allosteric regulation of the R domain to increase its affinity for the STAS domain, while, in the former, the stabilizers should be designed to work at the new interface to stabilize the interaction. An alternative would be to design PPI stabilizers to be used in association with modulators. In such a context, the modulators would improve the folding of CFTR, and the R domain as well, in the ER, thus restoring the interaction with SLC26A6, further facilitated by the use of PPI stabilizers (Figure 2).

Box 1. **Pharmacological targeting of protein-protein interactions.**

  Targeting PPIs has become a very attractive opportunity in drug discovery. As compared with canonical drugs, small molecules that modulate PPI are usually endowed with a higher specificity due to the structural and sequence diversity of protein–protein interfaces, and they can achieve efficacy even if the affinity for the target falls in the micromolar range because they do not compete with physiological ligands [51]. Although most of the efforts on small molecules targeting PPIs have been focused on inhibitors, the possibility to develop drug-like molecules able to stabilize the interaction among two or more protein partners has recently become an attractive possibility. A number of PPI stabilizers have been already identified [48,52], including some natural compounds and compounds validated as stabilizers in post hoc analyses (e.g., the mushroom toxin phalloidin that stabilizes actin filaments, [53]). Based on their mode of action, two different categories of PPI stabilizers can be identified: (i) molecules acting through allosteric stabilization, i.e., ligands that bind to an allosteric site of a specific target and induce a conformational change toward a structure showing higher affinity for a protein partner, and (ii) molecules that directly bind to an interfacial cage, thus improving complementarity and stabilizing the complex [48]. The second group is the most populated because the binding of two or more protein partners allows for a high level of specificity. A variety of techniques have been used to identify molecules that specifically target a PPI [54]. The premise for any approach is the detailed knowledge of the chemical-physical features of the contact surface area, and possibly of the geometry of the interaction between the partners, in order to select specific pockets that could be targeted by a putative drug-like molecule. Then, various screening strategies can be applied, including virtual screening, high throughput screening of compound libraries, fragment screening, and fragment-based drug design. Along with these strategies, a renewed interest has been focused on PPI stabilizers acting through covalent modification of specific residues because they could attain a higher duration of action and hold promise for the possible development of new drugs as well as new research tools [55]. Proteostasis-targeting chimera (PROTAC) molecules represent a striking example of successful use of this approach. They are able to induce the proteolytic degradation of a protein of interest through proximity effects because they are formed by a ligand that binds the target protein, connected to a ligand for ubiquitin ligase [56]. The covalent stabilization of PPIs can be achieved using different methods, such as tethering strategies through disulfide bond or imine bond formation, if known ligands of the protein of interest are not available, or proximity-enabled modification of known ligands through either ligand-based chemistry or the addition of a warhead usually formed by an aldehydic group that reacts with lysine residues of the protein partner [55].

## 4. Conclusions

In this perspective, we put forward the hypothesis that stabilizing the interaction between the STAS domain and the R domain of SLC26A6 and CFTR, respectively, in the presence or absence of modulators might help to restore ionic homeostatic regulation at extra-pulmonary sites affected by the disease. A background study on the biochemical and cellular features of the interaction between SLC26A6 and CFTR, and how this is affected by CFTR mutations as well as the concomitant use of HEMT, will be instrumental in the design of PPI stabilizers. We believe that the identification of such molecules might represent a potential therapeutic opportunity in the changing landscape of clinical management of PwCF, whose long-awaited improvement in pulmonary function and increased life expectancy now asks for due consideration of future co-morbidities in the aging patient population.

## Data Availability

Data sharing not applicable.

## References

[1] Shteinberg M., Haq I.J., Polineni D., Davies J.C. (2021). Cystic fibrosis. Lancet.

[2] Bell S.C., Mall M.A., Gutierrez H., Macek M., Madge S., Davies J.C., Burgel P.R., Tullis E., Castanos C., Castellani C. (2020). The future of cystic fibrosis care: A global perspective. Lancet Respir. Med..

[3] Jia S., Taylor-Cousar J.L. (2023). Cystic Fibrosis Modulator Therapies. Annu. Rev. Med..

[4] Ticona J.H., Lapinel N., Wang J. (2023). Future Comorbidities in an Aging Cystic Fibrosis Population. Life.

[5] Calthorpe R.J., Goodchild N., Gleetus V., Premakumar V., Hayee B., Elliott Z., Evans B., Rowbotham N.J., Carr S.B., Barr H. (2023). A grumbling concern: An international survey of gastrointestinal symptoms in cystic fibrosis in the modulator era. NIHR Open Res..

[6] Riordan J.R., Rommens J.M., Kerem B., Alon N., Rozmahel R., Grzelczak Z., Zielenski J., Lok S., Plavsic N., Chou J.L. (1989). Identification of the cystic fibrosis gene: Cloning and characterization of complementary DNA. Science.

[7] Blotas C., Ferec C., Moisan S. (2023). Tissue-Specific Regulation of CFTR Gene Expression. Int. J. Mol. Sci..

[8] Farinha C.M., Gentzsch M. (2021). Revisiting CFTR Interactions: Old Partners and New Players. Int. J. Mol. Sci..

[9] Chevalier B., Baatallah N., Najm M., Castanier S., Jung V., Pranke I., Golec A., Stoven V., Marullo S., Antigny F. (2022). Differential CFTR-Interactome Proximity Labeling Procedures Identify Enrichment in Multiple SLC Transporters. Int. J. Mol. Sci..

[10] Lostao A., Lim K., Pallares M.C., Ptak A., Marcuello C. (2023). Recent advances in sensing the inter-biomolecular interactions at the nanoscale—A comprehensive review of AFM-based force spectroscopy. Int. J. Biol. Macromol..

[11] Murabito A., Bhatt J., Ghigo A. (2023). It Takes Two to Tango! Protein-Protein Interactions behind cAMP-Mediated CFTR Regulation. Int. J. Mol. Sci..

[12] Schlessinger A., Zatorski N., Hutchinson K., Colas C. (2023). Targeting SLC transporters: Small molecules as modulators and therapeutic opportunities. Trends Biochem. Sci..

[13] Alper S.L., Sharma A.K. (2013). The SLC26 gene family of anion transporters and channels. Mol. Aspects Med..

[14] Ko S.B., Zeng W., Dorwart M.R., Luo X., Kim K.H., Millen L., Goto H., Naruse S., Soyombo A., Thomas P.J. (2004). Gating of CFTR by the STAS domain of SLC26 transporters. Nat. Cell Biol..

[15] Lohi H., Kujala M., Kerkela E., Saarialho-Kere U., Kestila M., Kere J. (2000). Mapping of five new putative anion transporter genes in human and characterization of SLC26A6, a candidate gene for pancreatic anion exchanger. Genomics.

[16] Waldegger S., Moschen I., Ramirez A., Smith R.J., Ayadi H., Lang F., Kubisch C. (2001). Cloning and characterization of SLC26A6, a novel member of the solute carrier 26 gene family. Genomics.

[17] Knauf F., Yang C.L., Thomson R.B., Mentone S.A., Giebisch G., Aronson P.S. (2001). Identification of a chloride-formate exchanger expressed on the brush border membrane of renal proximal tubule cells. Proc. Natl. Acad. Sci. USA.

[18] Wang Z., Petrovic S., Mann E., Soleimani M. (2002). Identification of an apical Cl^−^/HCO_3_^−^ exchanger in the small intestine. Am. J. Physiol. Gastrointest. Liver Physiol..

[19] Petrovic S., Wang Z., Ma L., Seidler U., Forte J.G., Shull G.E., Soleimani M. (2002). Colocalization of the apical Cl^−^/HCO3^−^ exchanger PAT1 and gastric H-K-ATPase in stomach parietal cells. Am. J. Physiol. Gastrointest. Liver Physiol..

[20] Petrovic S., Ma L., Wang Z., Soleimani M. (2003). Identification of an apical Cl^−^/HCO_3_^−^ exchanger in rat kidney proximal tubule. Am. J. Physiol. Cell Physiol..

[21] Jiang Z., Grichtchenko I.I., Boron W.F., Aronson P.S. (2002). Specificity of anion exchange mediated by mouse Slc26a6. J. Biol. Chem..

[22] Xie Q., Welch R., Mercado A., Romero M.F., Mount D.B. (2002). Molecular characterization of the murine Slc26a6 anion exchanger: Functional comparison with Slc26a1. Am. J. Physiol. Renal Physiol..

[23] Chernova M.N., Jiang L., Friedman D.J., Darman R.B., Lohi H., Kere J., Vandorpe D.H., Alper S.L. (2005). Functional comparison of mouse slc26a6 anion exchanger with human SLC26A6 polypeptide variants: Differences in anion selectivity, regulation, and electrogenicity. J. Biol. Chem..

[24] Clark J.S., Vandorpe D.H., Chernova M.N., Heneghan J.F., Stewart A.K., Alper S.L. (2008). Species differences in Cl^−^ affinity and in electrogenicity of SLC26A6-mediated oxalate/Cl^−^ exchange correlate with the distinct human and mouse susceptibilities to nephrolithiasis. J. Physiol..

[25] Wang Z., Wang T., Petrovic S., Tuo B., Riederer B., Barone S., Lorenz J.N., Seidler U., Aronson P.S., Soleimani M. (2005). Renal and intestinal transport defects in Slc26a6-null mice. Am. J. Physiol. Cell Physiol..

[26] Simpson J.E., Schweinfest C.W., Shull G.E., Gawenis L.R., Walker N.M., Boyle K.T., Soleimani M., Clarke L.L. (2007). PAT-1 (Slc26a6) is the predominant apical membrane Cl^−^/HCO3^−^ exchanger in the upper villous epithelium of the murine duodenum. Am. J. Physiol. Gastrointest. Liver Physiol..

[27] Freel R.W., Hatch M., Green M., Soleimani M. (2006). Ileal oxalate absorption and urinary oxalate excretion are enhanced in Slc26a6 null mice. Am. J. Physiol. Gastrointest. Liver Physiol..

[28] Jiang Z., Asplin J.R., Evan A.P., Rajendran V.M., Velazquez H., Nottoli T.P., Binder H.J., Aronson P.S. (2006). Calcium oxalate urolithiasis in mice lacking anion transporter Slc26a6. Nat. Genet..

[29] Ishiguro H., Namkung W., Yamamoto A., Wang Z., Worrell R.T., Xu J., Lee M.G., Soleimani M. (2007). Effect of Slc26a6 deletion on apical Cl^−^/HCO_3_^−^ exchanger activity and cAMP-stimulated bicarbonate secretion in pancreatic duct. Am. J. Physiol. Gastrointest. Liver Physiol..

[30] Greeley T., Shumaker H., Wang Z., Schweinfest C.W., Soleimani M. (2001). Downregulated in adenoma and putative anion transporter are regulated by CFTR in cultured pancreatic duct cells. Am. J. Physiol. Gastrointest. Liver Physiol..

[31] Ko S.B., Shcheynikov N., Choi J.Y., Luo X., Ishibashi K., Thomas P.J., Kim J.Y., Kim K.H., Lee M.G., Naruse S. (2002). A molecular mechanism for aberrant CFTR-dependent HCO_3_^−^ transport in cystic fibrosis. EMBO J..

[32] Lohi H., Lamprecht G., Markovich D., Heil A., Kujala M., Seidler U., Kere J. (2003). Isoforms of SLC26A6 mediate anion transport and have functional PDZ interaction domains. Am. J. Physiol. Cell Physiol..

[33] Gray M.A. (2004). Bicarbonate secretion: It takes two to tango. Nat. Cell Biol..

[34] Steward M.C., Ishiguro H., Case R.M. (2005). Mechanisms of bicarbonate secretion in the pancreatic duct. Annu. Rev. Physiol..

[35] Wang J., Wang W., Wang H., Tuo B. (2020). Physiological and Pathological Functions of SLC26A6. Front. Med..

[36] Yang D., Shcheynikov N., Zeng W., Ohana E., So I., Ando H., Mizutani A., Mikoshiba K., Muallem S. (2009). IRBIT coordinates epithelial fluid and HCO_3_^−^ secretion by stimulating the transporters pNBC1 and CFTR in the murine pancreatic duct. J. Clin. Investig..

[37] Song Y., Ishiguro H., Yamamoto A., Jin C.X., Kondo T. (2009). Effects of Slc26a6 deletion and CFTR inhibition on HCO_3_^−^ secretion by mouse pancreatic duct. J. Med. Investig..

[38] Simpson J.E., Gawenis L.R., Walker N.M., Boyle K.T., Clarke L.L. (2005). Chloride conductance of CFTR facilitates basal Cl^−^/HCO_3_^−^ exchange in the villous epithelium of intact murine duodenum. Am. J. Physiol. Gastrointest. Liver Physiol..

[39] Singh A.K., Riederer B., Chen M., Xiao F., Krabbenhoft A., Engelhardt R., Nylander O., Soleimani M., Seidler U. (2010). The switch of intestinal Slc26 exchangers from anion absorptive to HCO_3_^−^ secretory mode is dependent on CFTR anion channel function. Am. J. Physiol. Cell Physiol..

[40] Singh A.K., Sjoblom M., Zheng W., Krabbenhoft A., Riederer B., Rausch B., Manns M.P., Soleimani M., Seidler U. (2008). CFTR and its key role in in vivo resting and luminal acid-induced duodenal HCO_3_^−^ secretion. Acta Physiol..

[41] Ermer T., Nazzal L., Tio M.C., Waikar S., Aronson P.S., Knauf F. (2023). Oxalate homeostasis. Nat. Rev. Nephrol..

[42] Knauf F., Thomson R.B., Heneghan J.F., Jiang Z., Adebamiro A., Thomson C.L., Barone C., Asplin J.R., Egan M.E., Alper S.L. (2017). Loss of Cystic Fibrosis Transmembrane Regulator Impairs Intestinal Oxalate Secretion. J. Am. Soc. Nephrol..

[43] Moryousef J., Kwong J., Kishibe T., Ordon M. (2021). Systematic Review of the Prevalence of Kidney Stones in Cystic Fibrosis. J. Endourol..

[44] Wright J.F., Craig W.Y., Lucas F.L., Goldfarb D.S., Zuckerman J.B., Taylor E.N. (2021). Urinary stone disease prevalence and associations in cystic fibrosis. Urolithiasis.

[45] Lai S., Mazzaferro S., Mitterhofer A.P., Bonci E., Marotta P.G., Pelligra F., Murciano M., Celani C., Troiani P., Cimino G. (2019). Renal involvement and metabolic alterations in adults patients affected by cystic fibrosis. J. Transl. Med..

[46] Sato Y., Thomas D.Y., Hanrahan J.W. (2019). The anion transporter SLC26A9 localizes to tight junctions and is degraded by the proteasome when co-expressed with F508del-CFTR. J. Biol. Chem..

[47] Needham P.G., Goeckeler-Fried J.L., Zhang C., Sun Z., Wetzel A.R., Bertrand C.A., Brodsky J.L. (2021). SLC26A9 is selected for endoplasmic reticulum associated degradation (ERAD) via Hsp70-dependent targeting of the soluble STAS domain. Biochem. J..

[48] Chen S.Y., Zacharias M. (2023). What Makes a Good Protein-Protein Interaction Stabilizer: Analysis and Application of the Dual-Binding Mechanism. ACS Cent. Sci..

[49] Rosser M.F., Grove D.E., Chen L., Cyr D.M. (2008). Assembly and misassembly of cystic fibrosis transmembrane conductance regulator: Folding defects caused by deletion of F508 occur before and after the calnexin-dependent association of membrane spanning domain (MSD) 1 and MSD2. Mol. Biol. Cell.

[50] Fiedorczuk K., Chen J. (2022). Molecular structures reveal synergistic rescue of Delta508 CFTR by Trikafta modulators. Science.

[51] Thiel P., Kaiser M., Ottmann C. (2012). Small-molecule stabilization of protein-protein interactions: An underestimated concept in drug discovery?. Angew. Chem. Int. Ed. Engl..

[52] Zarzycka B., Kuenemann M.A., Miteva M.A., Nicolaes G.A.F., Vriend G., Sperandio O. (2016). Stabilization of protein-protein interaction complexes through small molecules. Drug Discov. Today.

[53] Holzinger A. (2022). Influencing the Actin Dynamics in Plant Cells by Jasplakinolide, Chondramides, Phalloidin, Cytochalasins, and Latrunculins. Methods Mol. Biol..

[54] Sheng C., Dong G., Miao Z., Zhang W., Wang W. (2015). State-of-the-art strategies for targeting protein-protein interactions by small-molecule inhibitors. Chem. Soc. Rev..

[55] Lucero B., Francisco K.R., Liu L.J., Caffrey C.R., Ballatore C. (2023). Protein-protein interactions: Developing small-molecule inhibitors/stabilizers through covalent strategies. Trends Pharmacol. Sci..

[56] Konstantinidou M., Li J., Zhang B., Wang Z., Shaabani S., Ter Brake F., Essa K., Domling A. (2019). PROTACs—A game-changing technology. Expert. Opin. Drug Discov..

